# Interoperability Between Health Information Systems: Dead End in Large-Scale Sharing of Health Data?

**DOI:** 10.2196/105089

**Published:** 2026-11-13

**Authors:** Fredrik Öberg, Sebastiaan Meijer

**Affiliations:** 1 Department of Perioperative Medicine and Intensive Care, Karolinska University Hospital, 171 76 Stockholm, Stockholm, Stockholm, 17176, Sweden, 46 706243114; 2 Department of Biomedical Engineering and Health Systems, KTH Royal Institute of Technology, Huddinge, Stockholm, Sweden

**Keywords:** medical informatics, interoperability, electronic health record, EHR, health information exchange, digital health, standards, semantics

## Abstract

This article provides a critical analysis of system-system interoperability (SSI) in the health data domain. By SSI, we mean the idea of connecting technical systems with each other to exchange operational data, directly or via intermediate platforms or infrastructures. We start by giving a brief history of some uses of the word “interoperability,” from the Institute of Electrical and Electronics Engineers (IEEE) original definition in 1991 to the Interoperable Europe Act of 2024. We thereby identify ambiguity and hidden assumptions in the definitions themselves. We then establish precise terminology to make a stringent analysis possible. Aided by a simple case of SSI from our own clinical practice and using a method of logical analysis, we look closely at (1) the assumption that the semantics in SSI can be handled by referring to external semantic standards, (2) the assumption that SSI is a feasible route to the safe exchange of operational data between systems in primary use, and (3) the challenges encountered in scaling SSI solutions to involve multidirectional data exchange, multiple data types, and multiple systems. Our first conclusion is that semantic interoperability, as defined by the Healthcare Information and Management Systems Society (HIMSS) in 2019, may not be a feasible route to stabilizing the real-world meaning of data exchanged in SSI solutions. The main reason is that referring to external standards cannot capture enough contextual information to correctly interpret health data remote from the primary context in which they were first registered. Our second conclusion is that SSI may not be a feasible route to the exchange of operational data between systems in primary use. Multiple unresolved problems are identified—problems that cannot be overcome by adding new components to existing systems, as proposed in, for example, the European Health Data Space (EHDS) legislation. Instead, a thorough redesign of each system’s internal architecture, data model, and user interface to align them with each other would be required. It is far from clear how such redesign would be practically achievable. Our third conclusion is that SSI may not be a scalable solution. In part, this is a consequence of the difficulties in using external standards to handle the semantics. Instead, careful negotiations and agreements about the exact meaning of each data type to be exchanged would be needed to guard against misinterpretation and, hence, risks in both primary and secondary use. We conclude that interoperability should be repositioned from being a taken-for-granted pathway to “better use of health data” to being an explicit object of critical inquiry. Further research should establish a commonly agreed analytical framework for evaluating suggested solutions. This framework must include stringent definitions of concepts and terminology, theoretical requirements, and practical aspects to be addressed in a concrete implementation.

## Introduction

It is estimated that health data account for more than 30% of the world’s data assets, with less than 3% of these data being used [1]. Initiatives to make better use of health data have been ongoing for decades, with notable examples from India, New Zealand, Australia, the United States, the United Kingdom, and the European Union (EU) [2]. We observe a common vision driving this global movement: health data should be readily reusable across organizational and country borders, respecting limits set by legislation and consents from the individuals to whom the data pertain, to create value for patients and health systems.

To our knowledge, virtually all major initiatives toward this vision are pivoting around the concept of interoperability. The current development of the European Health Data Space (EHDS), for instance, builds on joint standards and toolkits [3]. The implied assumption is that interoperability can provide the mechanism through which the intended outcomes will materialize. However, progress is slow despite the large resources spent [1,4,5]. This suggests that the problem is harder to solve than originally envisioned, calling for a deeper assessment of what is possible and what is not.

In this article, we critically assess the current status quo, starting with a brief historical account of the concept of interoperability itself and a breakdown of some central concepts into their conceptual components. We find implicit assumptions and ambiguity in the terms used, calling for more precise terminology. With this in place, we perform an analysis with the aim of promoting further research, informing political actors, and boosting the innovation agenda.

## Historical Perspective

In 1991, the Institute of Electrical and Electronics Engineers (IEEE) defined interoperability as “the ability of 2 or more systems or components to exchange information and to use the information that has been exchanged” [6]. Within the context of the IEEE, the word “system” in this era was understood to refer to technical systems. We will denote this as system-system interoperability (SSI). It is natural to interpret “exchange information” as referring to the exchange of data. There is an implicit yet critical assumption here: that exchanged data can be used by the receiving system without loss of meaning or function. In other words, with SSI, operational data from system A will become operational in system B—that is, available for its algorithms, user experience, interface, etc. This excludes read-only or referential access from the definition.

In 2005, the Healthcare Information and Management Systems Society (HIMSS) published its first definition of interoperability [7]. Its 2019 update [8], available on the HIMSS website [9], is probably the most widely used definition today. It defines interoperability as “the ability of different information systems, devices and applications (systems) to access, exchange, integrate, and cooperatively use data in a coordinated manner…to provide timely and seamless portability of information….” The focus remains on technical systems exchanging data that carry information; that is, we are still talking about SSI. The assumption of operational data is still there, now strengthened by the word “seamless.” As part of their 2019 update, HIMSS introduced the 4 “levels of interoperability”: foundational, structural, semantic, and organizational. The word “levels” may give the impression of something incremental, which we would argue is not the case. Rather, the levels should be regarded as necessary prerequisites in any meaningful implementation of SSI.

Semantic interoperability (SI) deserves some extra attention. The HIMSS definition reads as follows: “Semantic interoperability provides for common underlying models and codification of the data, including the use of data elements with standardized definitions from publicly available value sets and coding vocabularies, providing shared understanding and meaning to the user.” Looking closely, we see 2 parts: the first part is a normative statement about how SI must be achieved (by “common underlying models…standardized definitions from publicly available value sets and coding vocabularies”), and the second part is a statement about what will be provided (“shared understanding and meaning to the user”), but there is no formal definition of what SI actually is. However, a natural interpretation, and the one we will adopt in this paper, is that SI, as defined by HIMSS, is the prerequisite that shared data must denote the same concept in both sending and receiving systems. In the following, we will call this “de facto semantic interoperability” (DSI) to free the concept from any assumptions about how it is to be achieved.

By this breakdown, we see an implicit assumption in the HIMSS “definition,” stating that “shared understanding and meaning” can actually be achieved by “common underlying models….” This is not a trivial assumption. Moreover, the mechanism for this is not specified. In our interpretation throughout this paper, the HIMSS idea is that the systems involved in an SSI solution shall include, for each data type, a reference to a concept ID from some “common underlying model…” and that these IDs are to be carried over together with the data when transferred between systems. Obviously, the sender and receiver must agree on which “underlying model(s)” are to be used.

In 2017, the EU introduced the European Interoperability Framework (EIF) [10], where interoperability was defined as an “ability of organisations” to interact by means of “exchange of data between their ICT [information and communication technology] systems.” EIF also defined 4 levels of interoperability: legal, technical, semantic, and organizational. However, the levels are defined differently compared to HIMSS. The focus is now shifted from systems (as in IEEE/HIMSS) toward organizations. But note that the second half of this EIF definition is equivalent to interoperability as defined by IEEE, so interoperability between organizations is, by this definition, to be achieved by SSI between their ICT systems. These are, of course, 2 distinct entities, both of which are denoted by the word “interoperability,” blurring the distinction (see definitions below). And EIF, like IEEE, stresses the importance of SI and how to achieve it: “...taxonomies, controlled vocabularies, thesauri, code lists and reusable data structures/models are key prerequisites for achieving semantic interoperability” [11].

Building on EIF, the Interoperable Europe Act was adopted by the EU in 2024 [12]. Here, (cross-border) interoperability is defined as “ability of Union entities and public sector bodies to interact with each other across borders by sharing data, information and knowledge through digital processes in line with the legal, organisational, semantic and technical requirements related to such cross-border interaction.” As in EIF, exchange is still between organizations, but the sharing is expanded to include not only data but also information and knowledge. The term “digital processes” is not specified.

In the ISO 23903 standard, 7 levels of interoperability are listed [13]. In a paper from 2021, 27 types of interoperability were found in the literature [14]. As an example, the air forces of NATO (North Atlantic Treaty Organization) countries can be said to be more interoperable if they use the same fuel for their aircraft. A full discussion of all uses of the word “interoperability” is outside our scope. For this paper, it is sufficient to note the ambiguity and hidden assumptions discussed above.

## Some Definitions and Corresponding Goals

We will collectively refer to ontologies, vocabularies, taxonomies, terminologies, sets of definitions, etc, as “semantic standards.” For clarity of discussion, we will also introduce the term “system design standards” to refer to collections of data structures/models and templates to be used in implementation at the system level. Finally, from the viewpoint of health care actors and their technical systems, these are all considered “external standards” in the sense that they are set up and maintained by external authorities, organizations, or standardization bodies.

“Primary use” refers to use in a clinical context. For primary use, the vision stated in the *Introduction* section would substantially contribute to making health care processes and organizations interoperable in the sense of the EIF and the Interoperable Europe Act definitions.

“Secondary use” refers to research, follow-up, and policy settings. For secondary use, the vision stated in the *Introduction* section is about the ability to create insights from the aggregation of data from different health care settings for new and improved treatments and management. The most common analysis uses statistical methods and, more recently, AI, which in many cases does not require the same precision in individual patient data as in primary use. However, in many cases of secondary use, for example, when comparing caregivers’ performance, extreme precision is required, including deep contextual information.

SSI and DSI were defined in the *Historical Perspective* section above. Note that the term “system” covers any digital system unless otherwise specified. Electronic health record (EHR) systems and patients’ health apps, possibly connected to EHR systems by an application programming interface (API), are some examples.

## Objective

With the above preliminaries, we are now able to state our research question: to what extent can SSI take us closer to the vision stated in the *Introduction* section? To answer this question, we will only briefly touch on some specific solutions that have been suggested, since a complete account would not be possible and since this would be reverse logic—the burden of proof should be on the solutions themselves. Instead, our analysis will be generic. To aid us in the analysis, we will make use of a simple reference case from the Stockholm municipality area.

## The Stockholm Case: Interoperability in a Local Context

From 2017 onward, one of the authors initiated a project in which Karolinska University Hospital and 5 other hospitals were engaged to improve the flow of information in preoperative assessments. These 6 hospitals share a common EHR system. However, data from preoperative assessments were recorded by anesthetists not in the common EHR but in operation planning systems (OPSs), of which there was one for each hospital. In the project, the OPSs from the 6 hospitals were connected to the common EHR system so that medical data from preoperative assessments, a total of 19 data types, were sent from the OPSs to the common EHR. This was valuable not only when patients moved between hospitals but also within each hospital, where these data were made available to doctors and nurses not using the OPS. The project was successful, and the solution is still up and running.

The project’s planning phase involved discussions and workshops to make sure that the data types transferred from each OPS to the common EHR carried the same meaning for all actors involved. This required harmonization of both OPS system configurations (user experience, variables) and of system usage. In several instances, harmonization was not possible. A notable example is blood pressure, where the recorded figure could be patient-reported, measured by a nurse at a general practitioner clinic, measured by a surgeon, etc. Locally, in each hospital, that was fine because the context and procedures were known by everyone involved. However, the difference in workflows between hospitals was so large that harmonization was not practically possible, so blood pressure could not be included in the dataset shared. This would have implied a risk of interpreting one as the other, with patient safety issues as a result.

This is an example of SSI. The setting is primary use, there are very few actors and data types involved, and the data flow is only one-directional. With these limitations, the case will nevertheless serve us well in the discussion of larger-scale interoperability, since the difficulties encountered in this simple case can only be expected to increase at scale.

## Assumption 1: Interoperability by Standards

### Analytical Approach

We will now discuss 2 of the implicit assumptions found in the definitions of SSI and SI discussed in the *Historical Perspective* section above. The first one, derived from the HIMSS definition of SI, is what we call interoperability by standards. In this view, once data are documented in accordance with commonly agreed external standards, both their meaning (SI=HIMSS level 3) and their representation (HIMSS level 2) become sufficiently stabilized to allow safe and efficient reuse across systems and organizational boundaries, provided that HIMSS levels 1 and 4 are in place to handle technicalities and other necessary requirements.

### A Critical View on Interoperability by Standards

Research on classification and standardization demonstrates that external standards function as coordination mechanisms that require continuous local interpretation and adaptation, rather than as neutral carriers of universal meaning [15,16]. The Systematized Nomenclature of Medicine Clinical Terms (SNOMED CT)—the largest terminology-ontology in the field of health data globally—will be taken as an example. Looking at the Stockholm case above, would it have been of any help if that standard had been implemented in all the systems involved? Our answer to this question is negative because full contextual information, such as the operator, purpose, method, and process, is not part of this standard. System usage and, hence, the meaning of the data types involved could very well differ between hospitals despite identical IDs from semantic standards in the systems involved. We would still have needed the workshops. Moreover, adding context by referring to multiple concepts in postcoordination is a complex task [17], resulting in frequent coding errors [18], and we have not found any examples of this as part of an SSI solution. Similarly, system design standards would not have been sufficient because, even if the same data structures and templates had been used for data capture in all the OPSs, the context in which these templates were used would still not have been sufficiently described.

This is an example of something eloquently pointed out by Berg and Goorman [19] as early as 1999: medical data are dependent on deep contextual information to be correctly interpreted. Crucial elements may include subtleties like the training level of personnel involved, the reason for recording a specific measurement (or not), the method and equipment by which it was obtained, and the larger process of which it is a part. Another observation by Berg and Goorman is that the further away we move from the original context of data creation, the more contextual information will be needed to correctly interpret data, and capturing such rich metadata requires work. But incentives to do this work are lacking locally because context is already known there. It is therefore not obvious how this capturing can be made to reliably take place.

Moreover, and in line with these early observations, decades of research in science and technology studies and information systems challenge the assumption that meaning can be fully stabilized through formal representation. Bowker and Star’s [15] seminal work on classification systems further shows that standards inevitably foreground some perspectives while marginalizing others, and that their apparent universality masks substantial local adaptation work. From this standpoint, semantic standards do not carry meaning in isolation; rather, meaning is continuously reenacted in use. Even highly expressive standards such as SNOMED CT are intentionally context-agnostic, which makes them reusable across settings but also limits their capacity to encode clinically relevant situational information [20]. This tension between generality and contextual adequacy is not a technical shortcoming but a structural characteristic of external semantic standardization per se.

Furthermore, there is an important difference between often successful standardization in the technical domain (eg, USB connectors) and the proposed semantic standardization in the health domain. In the former, we adapt our practices to the standard because, if we do not, things will simply not work. In the latter, standardization will place constraints on how we are allowed to describe our practices, leaving the practices untouched. As a result, any given semantic standard will contain gaps— that is, practices that cannot be described within the standard. Filling these gaps is typically a multiyear process during which new gaps emerge as clinical knowledge and practice evolve [21]. However, reality cannot wait, so local ad hoc solutions will inevitably be created.

SNOMED CT is taken as an example. The reasoning can be extrapolated to any of the more than 1000 ontology standards that have been proposed in the health domain [22], including ISO 23903.

Content or transport standards like Health Level Seven (HL7) Fast Healthcare Interoperability Resources (FHIR) and HL7 Consolidated Clinical Document Architecture (C-CDA) help in structuring data for transport, and there are terminology bindings and contextual fields that may facilitate human understanding of what is transported. However, by the above arguments, this is not sufficient to guarantee DSI in an SSI solution.

It is unclear how the above issues could be managed so that external standardization could become a practically feasible means of stabilizing the real-world meaning of data types in SSI between health care systems in active use.

The above reasoning, focusing on EHR systems, is equally valid for any health data system, including patients’ health apps connected by APIs to other systems. Moreover, the critique, taking a case of primary reuse as a starting point, can be readily extrapolated to secondary use because the semantic issues are common to both settings, and misinterpreting data can be deleterious in either. The difference is a matter of time: in secondary use, it takes longer for errors to make any impact in the real world (eg, erroneous data leading to bad research, leading to incorrect treatment regimens, etc), whereas in primary use, errors will show up immediately in the care of individual patients. Taken together, these observations cast serious doubt on the validity of assumption 1, “Interoperability by standards,” in both primary and secondary use.

## Assumption 2: Shared Data Is Operational Data

### The Expected Role of Shared Data

This assumption, from both the IEEE and the HIMSS definitions, states that data shared through SSI will become operational in the systems involved. Imported data are not merely viewable or referential but are expected to be integrated into local data models, user interfaces, and algorithms, enabling automated processing, decision support, and the elimination of duplicated data entry in multiple systems. This is indeed a core feature of SSI, without which the concept would lose its meaning.

### A Critical View on the Operational Data Assumption

For this discussion, consider a case of multiple EHR systems in, say, a country. For the vast majority of data types, all EHR systems will have the capacity to record that data type in the context in which the system is used. Now, in an SSI solution, for shared data to become operational in the receiving system, they must be imported into its internal data structures, often requiring transformation. However, most contemporary EHR systems are designed to handle operational data that originate exclusively from their own user interfaces or from directly connected devices. Very few systems, if any, are designed to treat externally sourced data as first-class operational data. Treating externally sourced data as equivalent to locally produced data raises unresolved questions about provenance, trust, liability, and user comprehension. How should imported data be distinguished from data created in the system’s primary context? How should this distinction be presented in the user interface so that users understand what they are seeing? How can algorithms designed to act on locally generated data be extended or adapted to act safely on external data, potentially generated under different conditions? Empirical studies of EHR systems suggest that operational data are deeply coupled to the systems and contexts in which they are produced [23]. Data structures, user interfaces, and safety mechanisms are typically designed around locally generated data, and the introduction of externally sourced data can create new risks and unintended consequences [24]. Risk is further aggravated by the difficulties in transferring contextual information pointed out in the above section on standards. From a socio-technical perspective, these are not peripheral implementation issues but core design challenges that call into question the feasibility of operational data sharing across multiple systems per se.

For reasons evident from the above, these challenges cannot be resolved by adding external components to existing systems, such as the “European interoperability software component for EHR systems” of the EHDS legislation. Addressing them would require a thorough redesign of each system’s internal architecture, data model, and user interface to align them with each other, with considerable complexity and cost. It is far from clear that such redesign would be practically achievable or how such alignment could be maintained over time. The assumption of operational data therefore presupposes a degree of functional equivalence between local and external data that is rarely achieved in practice. Taken together, these considerations undermine assumption 2, “Shared Data Is Operational Data,” for EHR systems except in very limited and carefully designed local settings.

The above argument is restricted to primary use. In secondary use, the operational data assumption finds a parallel in “machine-actionable data,” a strongly emphasized component of the widely accepted FAIR (Findable, Accessible, Interoperable, and Reusable) principles [25], which have not proved easy to implement [26]. However, the argument cannot be extrapolated without a deeper discussion, which is outside our scope.

## The Scalability Challenge

As noted above, in the Interoperable Europe Act of 2024, interoperability was defined as a visionary state of affairs where actors “interact with each other across borders by sharing data, information and knowledge through digital processes….” We will now ask ourselves whether SSI could serve as the mechanism for this visionary state of affairs, similar to the vision stated in the *Introduction* section. If so, SSI would have to be scaled to include multiple systems, data types, and organizations in different countries using different languages. Is this possible?

The Stockholm case relied on a workshop-based approach, in which all actors discussed and negotiated the meaning and acceptable use of each data type. This approach is clearly not scalable to large numbers of systems and data types. Yet, as we saw, relying on external standards did not solve the underlying semantic issues either. This observed limitation can readily be extrapolated beyond this reference case. If external semantic and design standards cannot capture critical contextual information in a relatively simple local interoperability scenario, the difficulties will only increase in more complex settings, for both primary and secondary use.

Scholarly work has long emphasized that extensions from technical capabilities to their deployment at organizational levels are nontrivial [27,28]. From the latter perspective, such extensions cannot be reduced to data exchange alone but must be understood and analyzed as a situated accomplishment that depends on alignment across multiple layers of practice. Analyses of large-scale health IT programs showed that initiatives framed as technical integration projects repeatedly encounter failure modes rooted in organizational complexity, professional autonomy, and local context. These findings also resonate with earlier work in medical informatics emphasizing that health information systems are embedded in, and inseparable from, clinical work practices [29]. Health IT interventions operate within complex socio-technical systems, where outcomes emerge from the interaction of technology, professional practices, and institutional arrangements rather than from technical capabilities alone [27]. Empirical analyses of large-scale health IT programs further demonstrate that assumptions of scalability and transferability often break down when local organizational contexts are ignored [30].

Taken together, the above observations cast serious doubt on the scalability of SSI per se.

## A Nonexhaustive List of Further Requirements

For SSI to involve multidirectional operational data sharing between multiple systems, a number of further issues need to be handled. Authorizations and individual consents must be upheld across all systems involved. This would require some common handling of users, roles, and role-based permissions. Security must be maintained across the entire network of interconnected systems, whereby the overall level will be defined by the least secure participant. Multiple copies of the same data will be created as data are transferred. Determining the authoritative value and assigning responsibility for it become nontrivial. Finding information would require a common index spanning all systems in the network, which would be extremely complex and raise major integrity concerns. It must be ensured that all relevant data (eg, allergies, current prescriptions, and withdrawals) are reliably available when needed. Mechanisms for validating definitions, provenance, and data quality (who and what to trust) must be established. Mechanisms for data transfer (direct connections, intermediate platforms, push or pull) must be specified and established, including whether intermediate platforms are to store data or merely relay them and how availability and completeness are guaranteed in each case. Sharing of operational data between systems would have to involve near-real-time data transfer, which is technically possible but may prove extremely demanding or impossible to uphold at scale, considering the above-listed requirements.

## The Current Landscape

X-Road will be taken here as a well-known example infrastructure for data sharing. Provided by the Nordic Institute for Interoperability Solutions (NIIS), it is an open-source, centrally managed, distributed data exchange layer [31] used nationwide in Estonian public administration and adopted in other countries [32]. There are numerous unidirectional SSI solutions between Estonian authorities provided by the X-Road infrastructure [33]. However, when it comes to health data in Estonian EHR systems, a limited number of specified data types (eg, x-rays, lab results, and patient summaries) are transferred from the EHR systems to a national service, the eHealth record, where they may be accessed for viewing and analytics [34]. There are known issues with this service [35,36]. To our knowledge, and as indicated by the above-cited references, the X-Road infrastructure provides no SSI between Estonian EHR systems.

Health information exchange networks are widely used technical infrastructures for transferring data between caregivers, in much the same way as X-Road. While useful for many purposes, several issues and obstacles remain [37]. Our analysis in this paper is independent of how data is sent technically. Therefore, whether data are sent via a health information exchange network, via X-Road, or by other means is irrelevant to the validity of our argument. The identified issues will remain the same.

A discussion of AI is beyond the scope of this paper. AI could possibly be of help, for example, in adding contextual information that was not explicitly captured when data were created [38]. Note, however, that this contextual information must be expressed in some structure in which the semantics are handled, which takes us back to the problems with external standards. We see no way around this, with or without AI.

## Conclusions and Discussion

The above analysis suggests that (1) semantics in SSI cannot be handled solely by referring to external standards, (2) multidirectional SSI cannot be the bearing mechanism for operational data in a primary use setting, and (3) SSI will not scale to involve multiple actors, systems, and data types. These results cast serious doubt on whether SSI can be the mechanism to take us to the vision stated in the *Introduction* section. Therefore, advocates of the SSI approach must provide evidence, by real-world counterexamples to points (1) to (3) above or by valid theoretical reasoning, showing how the known challenges are to be met before proceeding with SSI-powered large-scale initiatives toward the vision.

Huber [39] shows how US interoperability regulations are explicitly mobilized to reconfigure health care markets toward platform logics and new forms of value extraction, while still relying on a “theory of change” that treats health data as quasi-raw, readily repurposable resources and assumes that opening infrastructures will automatically yield public value. Our findings resonate with and extend this critique: the European interoperability discourse similarly treats technical convergence and platform-ready infrastructures as self-evident routes to better care, innovation, and efficiency, while leaving unresolved the sociotechnical work, institutional redesign, and long-term governance required to make such promises credible [40]. If interoperability initiatives continue to function as what Huber calls “fulcrums” for data governance and platformization in health care, then interrogating and unsettling their underlying assumptions is not a purely conceptual exercise but a precondition for evaluating whose interests are advanced, which forms of value are prioritized, and which sociotechnical alternatives are crowded out.

We therefore suggest that future research agendas should treat interoperability itself as a contested sociotechnical object: one that must be specified, justified, and evaluated in context, in relation to concrete configurations of institutions, markets, and platforms. For policymakers, this implies that large-scale programs such as the EHDS should not proceed on the basis of an unexamined interoperability imaginary but should explicitly articulate and test their own theories of change, including how they will deal with context dependence, organizational diversity, and the possibility that full-scale operational data sharing may be both technically unattainable and normatively undesirable. In short, interoperability should be resituated from being a taken-for-granted pathway to “better use of health data” to being an explicit object of critical inquiry.

Moreover, given the ambiguity and implicit assumptions identified, concepts like interoperability and SI should be used with great care in communication or avoided altogether. The hidden assumptions we have found in the above analysis resonate with our own experience from taking part in several procurements of EHR systems and other large health data initiatives. The notion of SSI seems to be the most common interpretation of the word “interoperability” among policymakers and health care personnel. People expect that things will be “seamless,” as stated by HIMSS in its definition: interoperability will make data from remote caregivers’ EHR systems turn up in their own systems, alongside locally produced data and indistinguishable from it. Systems will be made to “talk to each other.” This will enable algorithms, including AI and precision medicine, to act on more complete data about a patient than is possible today. However, if SSI proves impossible at scale, as indicated by our analysis, the expected outcomes will not materialize by this route. Therefore, there is a risk that using the word “interoperability” and SI to describe goals and ambitions in large-scale health data initiatives will lead to expectations that cannot be met. We would advocate the use of more specific descriptive terms, such as referential access, read-only portals to health data from many caregivers, or sharing a few specific data types, such as patient summaries, x-rays, and e-prescriptions. Thereby, decisions and hope could be based on what can reasonably be expected, rather than on very unlikely visionary outcomes.

There might very well be novel technologies or paradigms that deserve to be studied as candidate solutions to take us to the vision. Knowledge graphs, data enclaves, or alternative sociotechnical designs might have a place. However, all proposed solutions should be evaluated against an analytical framework agreed on by the research community. This framework should include both practical and theoretical aspects against which candidate solutions could be evaluated for feasibility and expected outcomes. The terminology suggested and the conclusions drawn in this paper could serve as a starting point for this field of research.

## Abbreviations

API: application programming interface
C-CDA: Consolidated Clinical Document Architecture
CT: Systematized Nomenclature of Medicine Clinical Terms
DSI: de facto semantic interoperability
EHDS: European Health Data Space
EHR: electronic health record
EIF: European Interoperability Framework
EU: European Union
FAIR: Findable, Accessible, Interoperable, and Reusable
FHIR: Fast Healthcare Interoperability Resources
HIMSS: Healthcare Information and Management Systems Society
HL7: Health Level Seven
ICT: information and communication technology
IEEE: Institute of Electrical and Electronics Engineers
NATO: North Atlantic Treaty Organization
NIIS: Nordic Institute for Interoperability Solutions
OPS: operation planning system
SI: semantic interoperability
SSI: system-system interoperability
